# Paper mulberry fruit juice: a novel biomass resource for bioethanol production

**DOI:** 10.1186/s40643-021-00490-3

**Published:** 2022-01-08

**Authors:** Pleasure Chisom Ajayo, Mei Huang, Li Zhao, Dong Tian, Qin Jiang, Shihuai Deng, Yongmei Zeng, Fei Shen

**Affiliations:** 1 Institute of Ecological and Environmental Sciences, Sichuan Agricultural University, 211 Huimin Road, Wenjiang District, Chengdu, Sichuan People’s Republic of China; 2 Rural Environment Protection Engineering & Technology Center of Sichuan Province, Sichuan Agricultural University, Chengdu, Sichuan 611130 People’s Republic of China

**Keywords:** 1G feedstock, Ethanol conversion, Response surface methodology, Optimization, Nutrient screening

## Abstract

By way of broadening the use of diverse sustainable bioethanol feedstocks, the potentials of Paper mulberry fruit juice (PMFJ), as a non-food, sugar-based substrate, were evaluated for fuel ethanol production. The suitability of PMFJ was proven, as maximum ethanol concentration (56.4 g/L) and yield (0.39 g/g) were achieved within half a day of the start of fermentation, corresponding to very high ethanol productivity of 4.7 g/L/hr. The established potentials were further optimally maximized through the response surface methodology (RSM). At the optimal temperature of 30 °C, yeast concentration of 0.55 g/L, and pH of 5, ethanol concentration, productivity, and yield obtained were 73.69 g/L, 4.61 g/L/hr, and 0.48 g/g, respectively. Under these ideal conditions, diverse metal salts were afterward screened for their effects on PMFJ fermentation. Based on a two-level fractional factorial design, nutrient addition had no positive impact on ethanol production. Thus, under the optimal process conditions, and without any external nutrient supplementation, bioethanol from PMFJ compared favorably with typical sugar-based energy crops, highlighting its resourcefulness as a high-value biomass resource for fuel ethanol production.

**Graphical Abstract:**

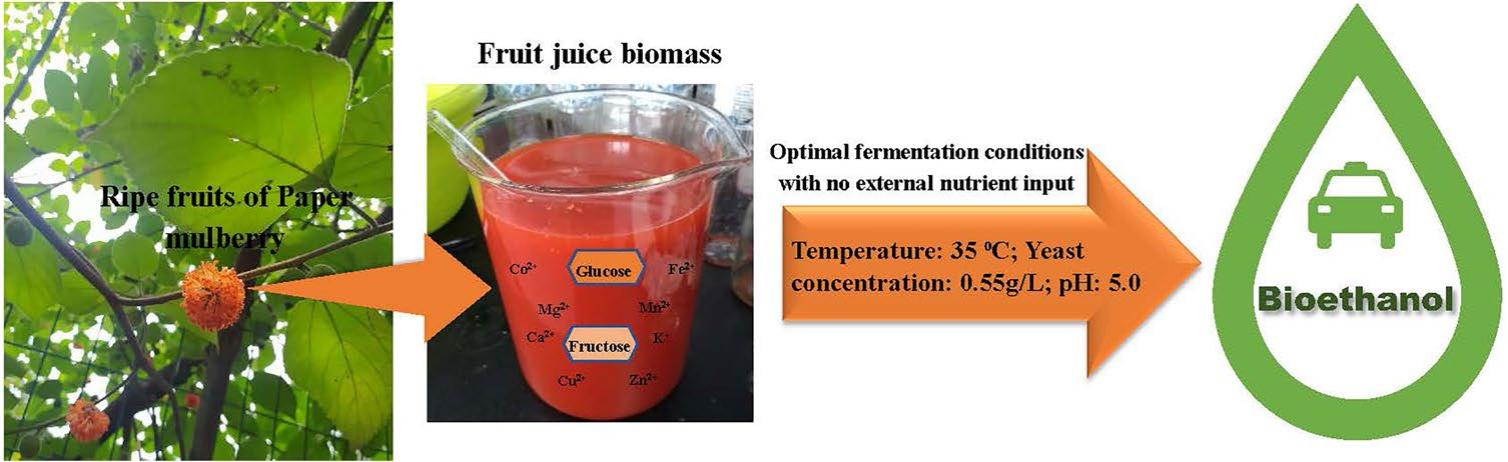

**Supplementary Information:**

The online version contains supplementary material available at 10.1186/s40643-021-00490-3.

## Introduction

At the United Nations General Assembly of September 22, 2020, China's president Xi Jinping committed his country to achieving carbon neutrality by 2060, in line with the Paris Agreement target of limiting global warming to 1.5 °C over this period (UN News 2020). Considering that the major source of carbon emissions is energy-related by burning of fossil fuels (Heede 2014), utilization of energy from biomass is a significant and sustainable strategy to achieving the goal of net-zero carbon emission (Zhang et al. 2021). Bioethanol, produced from the fermentation of sugars from different biomasses, is the most widely used and most demanded transport biofuel, accounting for approximately 71% of global biofuel production in 2019 (IEA 2020). It has numerous advantages over fossil-derived fuels, including its renewability, sustainability, and carbon–neutral nature (Micic and Jotanovic 2015). In view of these facts, many countries have implemented policies mandating that a set percentage of this liquid biofuel be blended with gasoline. Taking China as an example, the Central Government in 2017 stipulated that the mandatory use of E10 gasohol (gasoline containing 10% bioethanol) be expanded from 11 trial provinces to the entire nation by 2020 (Authur et al. 2017). This move is expected to contribute to a greener environment, a closer step to achieving the Paris Agreement goal, and a less dependence on crude oil. Meeting and keeping up with this national mandate thus require among other factors, the intensification of research efforts on the use of diverse feedstocks, coupled with efficient technological conversion processes (Zhang et al. 2021). However, as a developing country with very large human population, grain-based production of fuel ethanol in China is currently prohibited due to food security concerns (Dyk et al. 2016). This makes the utilization of non-food biomass more attractive, as it eliminates the food versus fuel debate, and further improves the economic competitiveness of bioethanol over fossil fuel.

Paper mulberry (*Broussonetia papyrifera* (L.) Vent.) is a non-food shrub or small tree that is indigenous to the Southwestern area in China, but now widely distributed in all of China, other Asian countries, the continent of Europe, as well as the Pacific Islands (Liao et al. 2014; Gonzalez-Lorca et al. 2015). Due to its aggressive invasiveness and wide adaptability to diverse ecologies, it is also gaining widespread dominance as an introduced specie in some African countries like Ghana and Uganda (Pe et al. 2016; Adigbli et al. 2018; Morgan et al., 2013; Yalley et al. 2020; Olupot 2022). Other attractive features of this tree include its strong germinating ability, rapid growth rate (height and diameter increments of 1 m and 1–2 cm per annum, respectively), high biomass yield, prolific regeneration capability, strong adaptability to stress conditions, and low management requirements (Thaiutsa et al. 2001; Peng et al. 2010; Xianjun et al. 2014). Paper mulberry (PM) trees are grown both in an agroforestry system and monoculture, where they serve multiple functions as fallow crop or soil improvers (Saito et al. 2009; Anning et al. 2018), intercrop specie (Thaiutsa and Puangchit 2001), afforestation trees (Kyereh et al. 2014), avenue/urban plantations (Maan et al. 2021), and as excellent raw materials for production of high-quality paper (Peng et al. 2019), textile (Peña-Ahumada et al. 2020), medicine (Park et al. 2017), and fabrication of modern bio-materials (Chen et al. 2017; Park et al. 2019; Kim et al. 2020). Relative to other components of PM tree such as the stem, stem bark, and roots, its fruits, which contain considerable amounts of soluble sugars, are not well investigated (Han et al. 2016). Being non-food fruits, they are mostly disregarded at their ripening period. Thus, they drop to the ground and rot, resulting in great loss of these sugar resources to the environment. This is especially critical considering its high fruit yield (mean of 11.24 tons per hectare) (Peng et al. 2010), and biannual fruiting and ripening patterns (Maan et al. 2020). The rich sugar content of PM fruit presents clues to its potential as a possible feedstock for use in first-generation (1G) bioethanol production. Apart from the research of Ding et al. (2016), that evaluated the use of its fruit juice as sugar baits for biological control of mosquitoes (*Culex pipiens pallens*), the utilization of the free sugars present in Paper mulberry fruit juice (PMFJ) remains largely unexplored.

Ethanol production using directly fermentable sugars (glucose, fructose, and sucrose) in juices is technically easier and more efficient, and produces higher ethanol titre compared to the use of starch or lignocellulosic biomass (Zabed et al. 2014; Cheng 2018). Yeast fermentation performance (as indicated by the concentration, amount, and rate of ethanol production) varies not only with the specie or strain involved, but also with the prevailing fermentation conditions including carbon source (feedstock), temperature, pH, and other growth factors. At sub or supra-optimal levels of these conditions, ethanol production can be inhibited as a result of impaired viability and vitality of yeast cells. Therefore, to greatly enhance the production and profitability of fuel ethanol, optimal levels of these conditions must be established (Mohd Azhar et al. 2017). Among various microorganisms for ethanol conversion, *Saccharomyces cerevisiae* (*S. cerevisiae*) cells are mostly employed in industrial process due to the facts that include but are not limited to their greater fermenting efficiency, and higher ethanol tolerance (Zabed et al. 2014). Using *S. cerevisiae*, varying process conditions of temperature, pH, and yeast concentrations have been reported for different sugar-based feedstocks because of the variations in biomass composition (Dodić et al. 2009; Hadeel et al. 2011; Giri et al. 2013; Nasidi et al. 2013; Thangadurai et al. 2014; Matharasi et al. 2018; Dular 2019). Nutrient composition of fermentation medium is another important factor influencing yeast performance for ethanol production (Tropea et al. 2016). In addition to nitrogen and phosphorus, the presence of metal ions (potassium, magnesium, zinc, calcium, copper, iron, cobalt, and manganese) and their interactions play very vital roles in yeast metabolic activities, and have been generally identified as critical nutrient factors affecting ethanol productivity, concentration, and yield, as well as increased tolerance of yeast to stress conditions (Rees and Stewart 1997; Pereira et al. 2010; Somda et al. 2011; Cao and Liu 2013). Sugar metabolism and ethanol production responses of yeast under external supplementation of these salt nutrients can differ, depending on the feedstock employed (Pereira et al. 2010; Cao and Liu 2013; Kelbert et al. 2015).

In this study, the potentials of PMFJ as a feedstock for bioethanol production were first evaluated. Then, the Response Surface Methodology (RSM) was further employed to optimize the fermentation conditions of temperature, yeast concentration, and pH with the aim of maximizing the ethanol production potentials of this substrate. Thereafter, under the optimal conditions established, external supplementation of nutrients in the forms of diverse salts was evaluated. Overall, this research thus opened up a pathway for the optimal bioconversion process of a new bioresource into ethanol, which is a contributory step toward meeting the need for a cleaner, cheaper, and sustainable energy.

## Materials and methods

### Biomass preparation

The ripe fruits of PM were harvested from the trees at the farm of Sichuan Agricultural University, Chengdu, China. The whole fruits were weighed and the orange-colored achenes (fruit part of interest) were separated from the seeds, and the core (green ball-like clusters of fleshy calyces). The separated achenes together with its juice were blended and sieved. The juice produced was recorded and immediately stored at − 18 ^°C^ pending analyses/processing.

### Yeast culture

Active dry yeast (*Saccharomyces cerevisiae*; Angel Yeast Co. Ltd., Yichang, China) was used for the bioconversion of juice sugars to ethanol. Using 50 mL synthesized medium (2 g/L yeast extract powder, 20 g/L protein, and 20 g/L glucose), 5 g of dry yeast was activated in a 250 mL flask for 2 h, at temperature of 35 °C and at 150 rpm. Thereafter, the activated yeast cells were separated from the nutrient medium by centrifugation at 5000 rpm for 5 min. The cells were then repeatedly washed using autoclaved distilled water at the same conditions of centrifugation, until a clear supernatant was obtained. The yeast slurry was dissolved in a certain volume of sterile water and the concentration determined, from which the required yeast amounts for fermentation were calculated accordingly.

### Batch fermentation experiments

To evaluate the potential of PMFJ as a feedstock for bioethanol production, preliminary batch fermentation was first performed. The pH of juice was adjusted to 6 using 2.5 mol/L NaOH, and autoclaved at 115 °C for 15 min. Yeast concentration of 6 g/L was inoculated into the substrate aseptically, and fermentation was carried out in an orbital shaker at 150 rpm and temperature of 35 °C for 96 h. This was repeated in triplicates. Samples were withdrawn at 12, 24, 48, 72, and 96 h, and centrifuged at 10,000 rpm for 5 min. The supernatants were stored at − 18 °C till subsequent analyses of residual sugar and ethanol concentrations.

To maximize established potentials of PMFJ for bioethanol production, juice fermentation conditions at varying levels of temperature, yeast concentration, and pH were performed for optimization. pH was carefully adjusted either with 2.5 mol/L NaOH or 2.5 mol/L HCl. Fermentation process was carried out as outlined above, but samples were this time withdrawn at shorter intervals (every 8 h for the whole incubation period of 80 h). Afterward, based on the optimized fermentation conditions, nutrient screening experiment was carried out to investigate the effects of diverse metal salts (KCl, MgSO_4_·7H_2_O, ZnSO_4_·7H_2_O, CaCl_2_·2H_2_O, CuSO_4_·5H_2_O, FeSO_4_·7H_2_O, CoCl_2_·6H_2_O, MnCl_2_·4H_2_O) on fermentation profile of PM fruit juice, with the aim of identifying the critical nutrient ions. These nutrient sources were chosen due to their relative low cost and ease of availability, bearing in mind their potential utilization in commercial fermentation operations.

### Analytical methods

The juice pH was directly measured using a pH meter (Shanghai Jingke Scientific Instrument Co., Ltd., China), while titratable acidity was determined by the method of Organisation for Economic Cooperation and Development (OECD 2018). Protein concentration was determined by the Bradford method (Kielkopf et al. 2020), using a Protein Quantification Kit (Nanjing Jiancheng Bioengineering Institute, China). Dinitrosalicylic acid (DNS) method was employed for the total reducing sugar (TRS) analysis (Miller 1959; Salari et al. 2019). The concentrations of individual glucose and fructose monosaccharides in the TRS were analyzed by a HPLC-RI (High-Performance Liquid Chromatography with a Refractive Index detector) equipped with the SH1011 column (Shodex, Showa Denko America, Inc., New York, USA). Operating conditions were: 0.05 mol/L H_2_SO_4_ as mobile phase, flow rate of 0.8 mL/min, and temperature of column and detector set at 50 °C and 60 °C, respectively. For the total soluble sugar determination, the fruit juice was first subjected to acid hydrolysis, to convert probably present sucrose to its monomeric sugars (Sewwandi et al. 2020). Thereafter, the total sugars were analyzed by DNS method. The concentrations of metallic nutrients in juice were determined by an Inductively Coupled Plasma-Optical Emission Spectrometer (Agilent 720 ICP-OES, Agilent Technologies, Inc., USA). The residual sugar and ethanol concentrations during fermentation were analyzed by the HPLC-RI as outlined above. From the detected ethanol concentration, ethanol yield (Eq. 1), productivity (Eq. 2), and fermentation efficiency (Eq. 3) were calculated

1 $$Y_{ps} = \, P/S,$$

*Y*_*ps*_, *P*, and *S* represent the ethanol yield (g/g), ethanol produced (g), and sugar consumed (g), respectively. Sugar consumed = initial sugar – residual sugar

2 $$Q_{p} = \, P/T.$$

*Q*_*p*_ symbolizes the ethanol productivity (g/L/h), and *P* and *T*, respectively, represent the maximum ethanol concentration (g/L), and fermentation time (h) at which it was obtained

3 $$F_{e} = \, Y_{ps} /0.511 \, \times \, 100;$$

*F*_*e*_ and *0.511* represent the fermentation efficiency (%), and maximum theoretical yield of ethanol from glucose, respectively.

### Experimental designs, statistical optimization, and analyses

The RSM is an effective statistical and predictive modeling approach, that optimizes multiple variables using minimum number of experimental runs. To maximize the potential of bioethanol production from PMFJ using *S. cerevisiae*, Box–Behnken design of RSM was used to optimize the three important fermentation conditions including temperature (20–40 °C), yeast concentration (0.5–2 g/L), and pH (4–6). The levels of each of these predictor variables were selected based on the preliminary fermentation of PMFJ, and the previous reports in literature (Zabed et al. 2014). Design-Expert software (Stat-Ease Inc., V 8.0.6., Minneapolis, USA) was used to generate the treatment combinations of 15 experimental runs including 3 central points, and was also utilized in data analyses. Ethanol concentration, and ethanol productivity, as important indicators of fermentation performance, were selected as the response variables for optimization. A second-order polynomial model was fitted to the obtained data of each response, to evaluate the individual and combined effects of the predictor variables on the response. Subsequently, numerical optimization was carried out, and the optimized fermentation conditions predicted by the model were validated experimentally.

For the nutrient screening, the regular Two-Level Fractional Factorial design was used to estimate the main, and two-factor interaction effects (2FI) of the nutrient variables. The two levels (low and high) of each nutrient variable in the unit of g/L were: A (KCl): 0.1 and 0.5; B (MgSO_4_·7H_2_O): 2.5 and 7.5; C (ZnSO_4_·7H_2_O): 0.01 and 0.09: D (CaCl_2_·2H_2_O): 0.2 and 0.8; E (CuSO_4_·5H_2_O): 0.025 and 0.125; F (FeSO_4_·7H_2_O): 0.01 and 0.09; G (CoCl_2_·6H_2_O): 0.001 and 0.03; H (MnCl_2_·4H_2_O): 0.001 and 0.02. Inexpensive nitrogen and phosphorus in the form of (NH_4_)_2_HPO_4_ was used as base nutrients at rate of 1.5 g/L. All nutrient sources and their corresponding rates were selected from literatures (Zhao et al. 2009; Pereira et al. 2010; Somda et al. 2011; Palma et al. 2012; Cao and Liu 2013; Tropea et al. 2016). Design-Expert software was used to generate 16 independent experimental runs, and was also employed in the analyses of obtained data. Alongside the generated runs, a sample with no nutrient addition was evaluated as a control.

## Results and discussion

### Composition of PMFJ and preliminary evaluation of its fermentability

The ripe fruits of PM were highly juicy, constituting almost half of the fresh fruit weight (Table 1). This confers on it a succulent and delicate structure (Maan et al. 2020), and a consequent increased susceptibility to microbial degradation of its sugars (Choosung et al. 2019). As typical of sugar-based biomasses, prompt harvest, swift juice extraction, and immediate storage of juice under appropriate conditions prior to fermentation are very important steps to ensure sugar preservation (Klasson and Boone 2021). The sum of glucose and fructose concentrations in juice (160.86 g/L) was almost the same with the total fermentable sugar (glucose, fructose, and sucrose; 161.7 g/L) (Table 1), indicating that the juice contained trace amount of sucrose sugar. Similarly, the total soluble sugar composition in ripe fruits of Mulberry (*Morus alba* L.), belonging to the same *Moraceae* family as Paper mulberry, was also reported to be made up of 80% of reducing sugars (Lee and Hwang 2017). A sugar concentration of 150–200 g/L is considered desirable in industrial bioethanol production (Zabed et al. 2014). The rich fermentable sugar in PMFJ is thus one of the indicators of its suitability as a high-value feedstock for commercial bioethanol production. Furthermore, as almost all of the fermentable sugars were in the form of monosaccharides (glucose and fructose), ethanol production might be initiated earlier due to the rapid passage of directly fermentable sugar monomers into the yeast cells, without prior hydrolysis in the yeast plasma membrane (Jasman et al. 2015). It is interesting to note that the concentration of fermentable sugar in PMFJ compares favorably with the raw juices of some notable sugar-based bioenergy crops, except sugar beets (Table 2). However, remarkable variations exist in their sugar composition, whereby unlike PMFJ, sucrose is the dominant saccharide in those sugar crops. The concentrations of minerals essential to yeast activities in PMFJ are shown in Table 1. The observed proportions of these ions are in agreement with an earlier study on the mineral composition of PM fruits (Sun et al. 2012). The nutrient ions present in PMFJ seemed sufficient to support a robust fermentation process, as all the essential metal ions were above the critical level required for yeast growth and metabolism (Walker 2014).

**Table 1:** Main composition of PMFJ

Constituents	Concentration	Constituents	Concentration
Juice content (g/kg fruit)	442.86 ± 0.73	*Mineral composition (mg/L)*	
pH	5.12 ± 0.01	K	2460.34 ± 5.2
Total titratable acidity (g/L)	1.60 ± 0.00	Ca	303.65 ± 1.7
Protein (mg/L)	235.18 ± 0.01	Mg	241.33 ± 3.3
*Sugar composition (g/L)*		Fe	25.40 ± 0.01
Total fermentable sugar	161.70 ± 1.04	Zn	2.96 ± 0.00
Glucose	83.72 ± 0.19	Cu	0.82 ± 0.00
Fructose	77.14 ± 0.19	Mn	0.61 ± 0.00
Sucrose	0.84 ± 0.00	Co	0.31 ± 0.00

*Each parameter value is the mean of triplicate values* ± *standard deviation*

**Table 2:** Fermentable sugars in PMFJ in comparison to other typical energy plants

Plant type	Total fermentable sugar (g/L)	Dominant fermentable sugar	References
Paper mulberry	161.7	Glucose and fructose; 99%	Current study
Sweet sorghum	96–170	Sucrose; 45–81%	(Luo et al. 2014; Barcelos et al. 2016; Rolz et al. 2019; Yue et al. 2021; Jebril et al. 2021a, 2021b)
Sugar cane	151–220	Sucrose; 83–91%	(Silva et al. 2017; Thammasittirong et al. 2017; Solís-Fuentes et al. 2019; Vu et al. 2020)
Sugar beet	104–270	Sucrose; 87–96%	(Gumienna et al. 2014; Marzo et al. 2019; Zicari et al. 2019; Bahrami et al. 2020; Garofalo et al. 2020; Hoffmann et al. 2021)

**Fig. 1:**
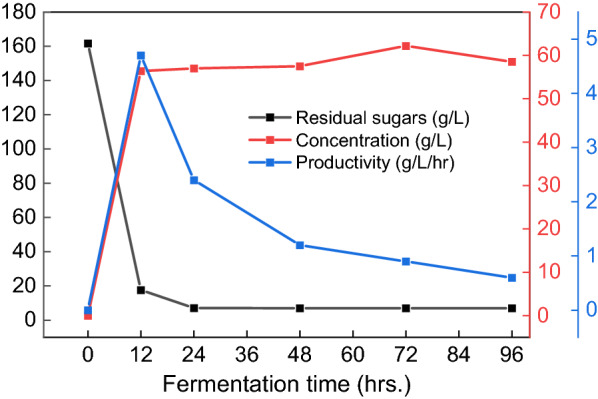
Yeast fermentation profile of PMFJ

To actually evaluate the potential of PMFJ as a viable feedstock for bioethanol production, preliminary batch fermentation was carried out using 6 g/L yeast concentration, for an incubation period of 96 h, and at temperature and pH of 35 °C and 6, respectively. At the first 12 h fermentation, the sugar concentration in the fermentation broth had dropped drastically from 161.7 to 17.6 g/L, corresponding to sugar consumption of 89.1% by the yeast (Fig. 1). Within the subsequent 12 h, a relatively lower amount of sugar was taken up. Afterward, no further uptake was observed due to depleting substrate concentration. With the high rate of sugar consumption, bioethanol was rapidly metabolized in the yeast cells and moved from the intracellular membranes into the fermentation broth; leading to an ethanol concentration of 56.4 g/L, produced at very high rate (productivity) of 4.7 g/L/hr within the first 12 h (Fig. 1). This concentration was above the minimum level (40 g/L) required for a cost effective down-stream ethanol distillation process (Chen et al. 2016). At subsequent periods, concentration remained relatively constant, indicating that stationary phase of ethanol production was already achieved within half a day of the start of fermentation.

The presence of metal ions (such as potassium, magnesium, zinc, calcium, manganese, iron, cobalt, and copper) in fermentation medium play a crucial role in yeast metabolism, as they primarily act as co-factors for a large number of enzymes involved in bioethanol production (Walker and Walker 2018). The inherent yeast-essential mineral nutrients in PMFJ were all above the threshold level required, which could have resulted in its excellent fermentability, in terms of ethanol concentration and productivity. Additionally, the quick rate of sugar uptake suggested the absence of components in the sugar substrate that could prove inhibitory to yeast cells, such as some toxic ions (Walker 2014). The rapid rate of sugar uptake by the yeast also seemingly confirmed our earlier speculation that the movement of sugars into the yeast cells would be faster with the directly fermentable glucose and fructose sugars. This could be because there would be no prior sucrose hydrolysis into its monomers in the yeast plasma membrane (Jasman et al. 2015).

Bioethanol yield represents the amount of ethanol produced relative to the amount of sugar consumed. A higher yield would suggest that a greater portion of the total consumed sugar was actually incorporated into the metabolic pathway of producing the desired product (bioethanol). Based on stoichiometric mass balance, the maximum theoretical ethanol yield from 1.0 g of consumed fermentable sugar monomer is 0.51 g. On a practical basis though, some sugars will expectedly be used up in some side reactions necessary for ethanol synthesis. Therefore, bioethanol yield corresponding to at least 90% of the maximum theoretical yield (fermentation efficiency) is seen as being good in practice (Zabed et al. 2014). The obtained bioethanol yield of 0.39 g/g from fermentation of PMFJ was equivalent to 76.5% of the maximum theoretical yield, which fell short of the minimum level. In a subsequent evaluation, the fermentation performance of this novel biomass resource was further improved through optimization of process conditions.

### Optimization of bioethanol production from PMFJ

The RSM is one of the experimental models for obtaining optimum settings for a range of factors affecting response variable(s) of interest. Three fermentation factors each at three levels (low, midpoint, and high) were evaluated using Box–Behnken design of RSM to optimize ethanol concentration and productivity. Unlike the preliminary study, yeast concentration was reduced to 0.5–2 g/L for the optimization, considering the facts of sugar substrate and its rapid uptake, as well as the input cost. The maximum temperature was extended to 40 °C, with minimum of 20 °C, while the pH values ranged from 4 to 6. Samples were withdrawn every 8 h for a whole duration of 80 h. At 16 h, most of the treatment combinations had achieved stationary phases of sugar uptake and ethanol production. Therefore, data collected at this time-point were used for evaluation.

#### Responses of ethanol concentration and productivity to fermentation conditions

With the use of quadratic polynomial function, the relationships of ethanol concentration and productivity with the three fermentation conditions of temperature, yeast concentration, and pH were described (Eqs. 4 and 5)

4 $$\begin{aligned} Y_{{\text{Ethanol concentration}}} = & \, 71.12 + 29.19X_{1} {-}0.20X_{2} + \, 1.31X_{3} {-}1.68 \, X_{1} X_{2} \\ {-} \, 0.59 \, X_{1} X_{3} {-}1.21 \, X_{2} X_{3} {-}28.43X_{1}^{2} {-}0.63X_{2}^{2} + 1.18X_{3}^{2} \\ \end{aligned}$$

5 $$\begin{aligned} Y_{{\text{Ethanol productivity}}} = & \, 4.44 \, + \, 1.19X_{1} + \, 0.02X_{2} + \, 0.11X_{3} {-} \, 0.18X_{1} X_{2} \\ {-} \, 0.08 \, X_{1} X_{3} {-}0.08 \, X_{2} X_{3} {-}1.14X_{1}^{2} - 0.04X_{2}^{2} + \, 0.08X_{3}^{2} . \\ \end{aligned}$$

The analysis of variance (ANOVA) for the quadratic models of ethanol concentration, and productivity were highly significant, as *p* < 0.0001, and *p* = 0.0001, respectively (Table 3). This indicated that the models for the regression terms were adequate, and a higher order model would not be needed. As seen in the R-square values of the models, more than 99% of variations in the both responses could be explained by the factors of fermentation conditions, reflecting the model reliability. The models for the two responses passed the lack of fit test, as *P* values were higher than 0.05, showing that the experimental data fitted well to the model, and could suitably be used for prediction. The less than 5% coefficient of variation (CV) was a proof of the reproducibility and reliability of experimental data. Based on the *p* values of the three considered fermentation conditions, only temperature had highly significant main linear effects on the two dependent variables (Table 3). There were positive responses of ethanol concentration and productivity to increases in temperature, with linear coefficients of 29.19, and 1.19, respectively (Eqs. 4 and 5). None of the interaction effects of the fermentation factors on the both responses were significant. This signaled that the remarkable impact exhibited by temperature basically remained the same, irrespective of the prevailing conditions of yeast concentration and pH within the considered ranges (Additional file 1: Fig. S1). Bioethanol concentration and productivity exhibited no significant quadratic responses to yeast concentration and pH, but had a highly significant curve relationship with fermentation temperature. The quadratic impact of temperature caused a significant reduction in the responses, which was indicated by the negative values of the coefficients in the polynomial functions. Therefore, the optimal region for each dependent variable in response to temperature was a maximum rather than minimum (i.e., the curvature is convex) (Additional file 1: Fig. S1). This meant that while bioethanol concentration and productivity initially responded positively to temperature increase, a further unit increase above the optimal level would result in significant reductions at magnitudes of − 28.43 and − 1.14, respectively (Eqs. 4 and 5).

**Table 3:** ANOVA for the quadratic models of ethanol concentration and productivity

Sources of variance	Ethanol concentration			Ethanol productivity		
	Sum of square	F value	*p* value	Sum of square	F value	*p* value
Model	9885.36	226.42	< 0.0001	16.47	69.87	0.0001
Temperature—X_1_	6818.78	1405.66	< 0.0001	11.28	430.61	< 0.0001
Yeast conc.—X_2_	0.30	0.06	0.8122	0.00	0.17	0.6953
pH—X_3_	13.73	2.83	0.1533	0.09	3.45	0.1225
X_1_X_2_	11.39	2.33	0.1876	0.13	4.81	0.0798
X_1_X_3_	1.39	0.28	0.6151	0.03	1.04	0.3548
X_3_X_4_	5.86	1.21	0.3219	0.02	0.86	0.3966
X_1_^2^	2985.41	615.43	< 0.0001	4.81	183.70	< 0.0001
X_2_^2^	1.47	0.30	0.6062	0.01	0.27	0.6225
X_3_^2^	5.14	1.06	0.3505	0.02	0.92	0.3813
Lack of fit	20.87	4.12	0.2016	0.12	5.37	0.1610
R^2^	0.9976			0.9921		
CV	3.92			3.85		

The observed and predicted values of ethanol concentration and productivity as a function of fermentation conditions are shown in Table 4. The observed values varied from 9.61 to 76.51 g/L, and 1.72 to 4.78 g/L/hr, respectively. Based on the amount of sugar consumed, this corresponded to ethanol yields of 0.18–0.51 g/g (35–100% of the maximum theoretical yields or fermentation efficiencies). The predicted values of the responses by the model matched closely with the actual experimental data obtained, as revealed by the very small residual values. Yeast concentration and pH within the evaluated ranges were not critical process conditions influencing ethanol titre and rate of formation. Though generally, there were slight negative responses at lower values of these predictor factors. At the same conditions of yeast concentration and pH, an increase in temperature above 20 °C resulted in significant improvements in ethanol concentration. There were increases from 16.21–72.31 g/L (runs #4 vs #10), 14.12–72.70 g/L (runs #11 vs #14), 9.61–72.47 g/L (runs #2 vs #8), and 15.00–71.14 g/L (runs #7 vs #13). The same trend was also observed in the rate of ethanol production, from 2.36 to 4.52 g/L/hr (runs #4 vs #10), 2.08 to 4.54 g/L/hr (runs #11 vs #14), 1.72 to 4.53 g/L/hr (runs #2 vs #8), and finally from 2.34 to 4.44 g/L/hr (runs #7 vs #13). These tremendous increases matched well with the rate of sugar consumption. At just 16 h fermentation, stationary phase of sugar uptake had been achieved by most runs involving a temperature of above 20 °C. On the other hand, sugar metabolism was really slow at 20 °C, resulting in a much later attainment of stationary phase at 32–40 h (Additional file 1: Table S1). It should also be noted that irrespective of the fermentation condition, residual fructose concentrations in the fermentation broth were remarkably higher than residual glucose at every sampling time (Additional file 1: Table S1). Though both carbon sources were simultaneously metabolized, the yeast cells showed higher preference for glucose assimilation relative to fructose, as the rate of glucose uptake was noticeably faster. This fact is well established in literatures (Pinu et al. 2014; Weinhandl et al. 2014; Kayikci and Nielsen 2015; Díaz-Nava et al. 2017; Endoh et al. 2021).

**Table 4:** Actual and predicted values for ethanol concentration (g/L) and productivity (g/L/hr) based on Box–Behnken design

Runs	Codes			Ethanol concentration			Ethanol productivity		
	X_1_	X_2_	X_3_	Observed	Predicted	Residual	Observed	Predicted	Residual
1	30	2	4	69.25	71.38	− 2.13	4.33	4.47	− 0.14
2	20	0.5	5	9.61	11.38	− 1.77	1.72	1.87	− 0.15
3	30	0.5	6	76.51	74.39	2.12	4.78	4.64	− 0.14
4	20	1.25	6	16.21	16.57	− 0.36	2.36	2.38	0.00
5	30	1.25	5	69.82	71.12	− 1.30	4.36	4.44	− 0.08
6	30	1.25	5	71.12	71.12	0.00	4.44	4.44	− 0.00
7	20	2	5	15.00	14.35	0.65	2.34	2.27	0.07
8	40	0.5	5	72.47	73.13	− 0.65	4.53	4.60	0.09
9	30	1.25	5	72.42	71.12	1.30	4.53	4.44	− 0.07
10	40	1.25	6	72.31	73.78	− 1.47	4.52	4.59	0.08
11	20	1.25	4	14.12	12.77	1.47	2.08	2.01	0.15
12	30	0.5	4	69.64	69.35	0.29	4.35	4.28	− 0.00
13	40	2	5	71.14	69.38	1.76	4.44	4.29	− 0.08
14	40	1.25	4	72.70	72.34	0.36	4.54	4.55	− 0.01
15	30	2	6	71.28	71.58	− 0.29	4.46	4.54	− 0.08

Temperature has been implicated as the top factor having strong impact on fermentation performance by yeast (Lin et al. 2012; Zabed et al. 2014; Bhadana and Chauhan 2016; Mohd Azhar et al. 2017). For one, it affects fluidity of yeast membranes; subsequently impacting on the passage of solutes into and out of cells (Zabed et al. 2014). Over a 168 h incubation, Lin et al. (2012) observed that increasing the temperature from 10 to 20 °C, and then up to 30 °C shortened the exponential growth period of yeast cells to 120 and 48 h, respectively. It was then concluded that the quicker onset of stationary phase was initiated as a result of increased cell division and metabolic activities. Similarly, at each evaluated temperature level in this current study, a comparison of the residual sugar in fermentation broth with the corresponding bioethanol concentration and rate of production revealed a strong inverse relationship (Additional file 1: Fig. S2). The poor fermentation performance at the low temperature of 20 °C was therefore a consequence of reduced uptake of fermentable sugar molecules for conversion into bioethanol, owing to a decreased yeast metabolic rate. With increase in temperature beyond 20 °C and up to a point, sugar uptake was improved tremendously (varying from 89.5–95.2% consumption). Bioethanol was rapidly metabolized in the yeast cells, and moved from within the cells into the fermentation broth leading to high ethanol concentration, and attainment of stationary phase at just the 16 h incubation. However, much higher increase in temperature up to 40 °C presented a stress factor to yeast cells, which led to significant reductions in ethanol production. There was also a corresponding increase in the amount of residual sugar, indicating inhibited substrate uptake (Additional file 1: Table S1). The metabolic and physical mechanisms behind this inhibition was reported to include inactivation of regulatory enzymes, denaturation of yeast ribosomes, and change in fluidity of yeast membranes; which hindered inter- and intracellular solute movement, resulting in the accumulation of toxins in yeast cells, and reduced uptake of the much needed carbon substrate (Walker 1998). It is worth stating that even at extreme temperature condition of 40 °C, the concentrations of bioethanol from PMFJ (71.14–72.70 g/L) were still above the minimum requirement (40 g/L) for industrial fermentation, and the maximum productivity (4.52–4.54 g/L/hr) exceeded many reported values in literatures from the fermentation of other sugar substrates (Additional file 1: Table S2). This could be related to the abundant availability of minerals in the juice, especially magnesium ion. This mineral exerts a membrane protective effect on yeast cells, enabling an enhanced ethanol production even under temperature stress (Eardley and Timson 2020; Walker and Basso 2020).

Varying literature reports exist with respect to the influence of yeast concentration on bioethanol production. According to the findings of Matharasi et al. (2018) on batch fermentation of Banana fruit waste, increasing yeast concentration levels from 1 to 5% progressively improved bioethanol concentration significantly. Conversely, in a review of several studies on yeast bioethanol production, Mohd Azhar et al. (2017) reported that while higher yeast concentration had no effect on the final ethanol titre, it markedly influenced the rate of ethanol formation (productivity). This was as a result of the reduction in incubation period, due to more rapid sugar uptake by the large yeast cells population. In an optimization modeling of bioethanol production from sweet sorghum juice, Luo et al. (2014) noted no significant effect on both the final ethanol titre and ethanol productivity, under the evaluated yeast concentrations of 0.5–2 g/L. Similarly, increase in the yeast concentrations within the range used in this current research (0.5–2 g/L) had no significant effects on ethanol concentration and productivity. Even if higher amounts of yeast cells were used, the possibility of observing a significant effect was not justifiable. This is in consideration of the fact that during the preliminary investigations to evaluate fermentability of PMFJ, the obtained ethanol productivity and concentration using 6 g/L of yeast cells (Fig. 1) were, respectively, at par with, and even lower than that obtained under the reduced yeast levels used in the optimization study, at similar temperature and pH conditions (Table 4). Therefore, the excellent performance of PMFJ even at very low yeast concentrations could be attributed to the substrate-related factors. These included its rich essential mineral nutrients’ status, the fermentable sugars being mostly composed of glucose and fructose monosaccharides, as well as the absence of any yeast-inhibitory factor in the juice that could impair cells activities.

The H^+^ concentration (pH) of the fermentation broth affects nutrients permeability into the yeast cells, which in extension influences yeast metabolism, ethanol production, and by-product formation (Lin et al. 2012; Zabed et al. 2014) In our study, while there were negative responses of ethanol concentration, and productivity to low pH value of 4, the impact of pH was not significant.

#### Numerical optimization and validation of model prediction

**Fig. 2:**
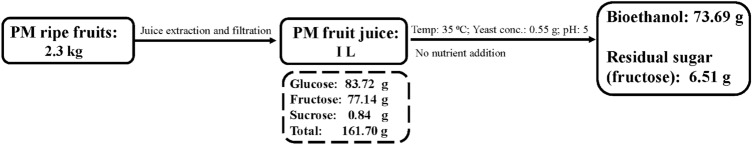
Mass balance of ethanol production from PMFJ under the optimal process conditions

Optimization was achieved based on the criteria of maximizing bioethanol concentration and productivity, while keeping the temperature, yeast concentration, and pH in range settings. The optimized fermentation conditions predicted by the model were temperature of 35 °C, yeast concentration of 0.55 g/L, and pH of 5.0, which would result in ethanol concentration, and productivity of 79.14 g/L, and 4.78 g/L/hr, respectively. These optimal fermentation conditions predicted by the model were verified by performing the corresponding experiment in triplicates. The mean responses of ethanol concentration, and productivity subsequently obtained (16 h fermentation) were all within the 95% confidence interval (Table 5), confirming the model prediction. The mass balance of ethanol production under these optimal process conditions is displayed in Fig. 2.

**Table 5:** Confirmation of the optimized fermentation conditions predicted by the model

Responses	Predicted value	95% CI^*a^	Observed value
Concentration (g/L)	79.14	72.47–85.83	73.69^*b^
Productivity (g/L/hr)	4.78	4.29–5.27	4.61

^*^^a^ Confidence interval

^*b^Based on the amount of sugar consumed, this represented an ethanol yield of 0.48 g/g (94% of the maximum theoretical yield)

With the use of *S. cerevisiae* in batch fermentation, different optimal process conditions have been reported for several sugar-based feedstocks (Additional file 1: Table S2). While the ideal temperature and pH established for the fermentation of PMFJ were well within the ranges generally reported in literatures, the optimal yeast concentration differed greatly. Interestingly, even at relatively very low yeast concentration, bioethanol production from PMFJ compared favorably with some notable sugar-based energy plants, and even exceeded most other 1G feedstocks, which can boost its economic suitability by way of reductions of process time and cost.

### Nutrient screening based on a two-level fractional factorial design

The high mineral contents of PMFJ provided a theoretical basis for the assumption that fermentation process could be efficiently sustained without external nutrient addition. However, to confirm this in practical terms, yeast-essential macro- and micronutrient ions supplied by diverse salts were supplemented to the fermentation medium, to check for the possibility of further improvement in final ethanol concentration. Fermentation operations were carried out based on the previously optimized temperature, yeast concentration, and pH conditions, and a sample with no added nutrient was used as control. Maximum ethanol concentration was achieved at 16 h in all runs. Table 6 displays the actual and predicted values of final ethanol concentration based on a two-level fractional factorial design, with the observed values ranging from 65.55 to 83.83 g/L. As shown in Table 7, the employed factorial model in the ANOVA was highly significant (*p* < 0.01), indicating that the model was suitable to assess the response of ethanol concentration to nutrient supplementation. Furthermore, almost all the variabilities observed in the response were explainable by the nutrient factors (*R*^2^ = 0.9830), and the low CV value (2.18) indicated reliability of experimental data.

**Table 6:** Actual and predicted values of final ethanol concentration based on a two-level fractional factorial design

Runs	KCl	MgSO_4_	ZnSO_4_	CaCl_2_	CuSO_4_	FeSO_4_	CoCl_2_	MnCl_2_	Ethanol concentration (g/L)		Residual
									Observed	Predicted	
1	0.5	2.5	0.09	0.8	0.025	0.01	0.03	0.001	78.91	78.08	0.84
2	0.5	2.5	0.01	0.8	0.125	0.09	0.001	0.001	65.72	65.28	0.45
3	0.1	2.5	0.09	0.8	0.125	0.01	0.001	0.02	70.19	71.11	− 0.92
4	0.1	7.5	0.09	0.8	0.025	0.09	0.001	0.001	82.26	83.54	− 1.28
5	0.5	7.5	0.09	0.2	0.125	0.01	0.001	0.001	66.34	66.70	− 0.36
6	0.1	7.5	0.01	0.2	0.125	0.09	0.001	0.02	75.00	74.17	0.84
7	0.5	7.5	0.01	0.2	0.025	0.09	0.03	0.001	72.67	73.59	− 0.92
8	0.5	7.5	0.09	0.8	0.125	0.09	0.03	0.02	65.55	64.35	1.20
9	0.5	2.5	0.09	0.2	0.025	0.09	0.001	0.02	78.38	78.37	0.00
10	0.5	2.5	0.01	0.2	0.125	0.01	0.03	0.02	69.71	70.99	− 1.28
11	0.1	2.5	0.09	0.2	0.125	0.09	0.03	0.001	74.44	74.37	0.07
12	0.1	7.5	0.09	0.2	0.025	0.01	0.03	0.02	81.54	81.10	0.44
13	0.1	2.5	0.01	0.2	0.025	0.01	0.001	0.001	83.83	82.63	1.20
14	0.5	7.5	0.01	0.8	0.025	0.01	0.001	0.02	78.54	78.47	0.07
15	0.1	7.5	0.01	0.8	0.125	0.01	0.03	0.001	82.02	82.01	0.00
16	0.1	2.5	0.01	0.8	0.025	0.09	0.03	0.02	71.79	72.14	− 0.36
Control	0	0	0	0	0	0	0	0	78.04

**Table 7:** Analysis of variance for selected factorial model of ethanol concentration

Sources	Sum of square	F value	*P *value	Standardized effect
Model	569.08	20.97	0.0049	
A-KCl	127.97	51.88	0.0020	− 5.66
B-MgSO_4_	7.49	3.04	0.1563	1.37
C-ZnSO_4_	0.1743	0.0707	0.8035	− 0.21
D-CaCl_2_	3.00	1.22	0.3319	− 0.87
E-CuSO_4_	217.19	88.04	0.0007	− 7.37
F-FeSO_2_	39.91	16.18	0.0158	− 3.16
G-CoCl_2_	0.8236	0.3338	0.5944	− 0.45
H-MnCl_2_	15.00	6.08	0.0693	− 1.94
AB	56.96	23.09	0.0086	− 3.77
AE	34.25	13.88	0.0204	− 2.93
AH	66.30	26.88	0.0066	4.07
R^2^	0.9830			
CV	2.10			

**Fig. 3:**
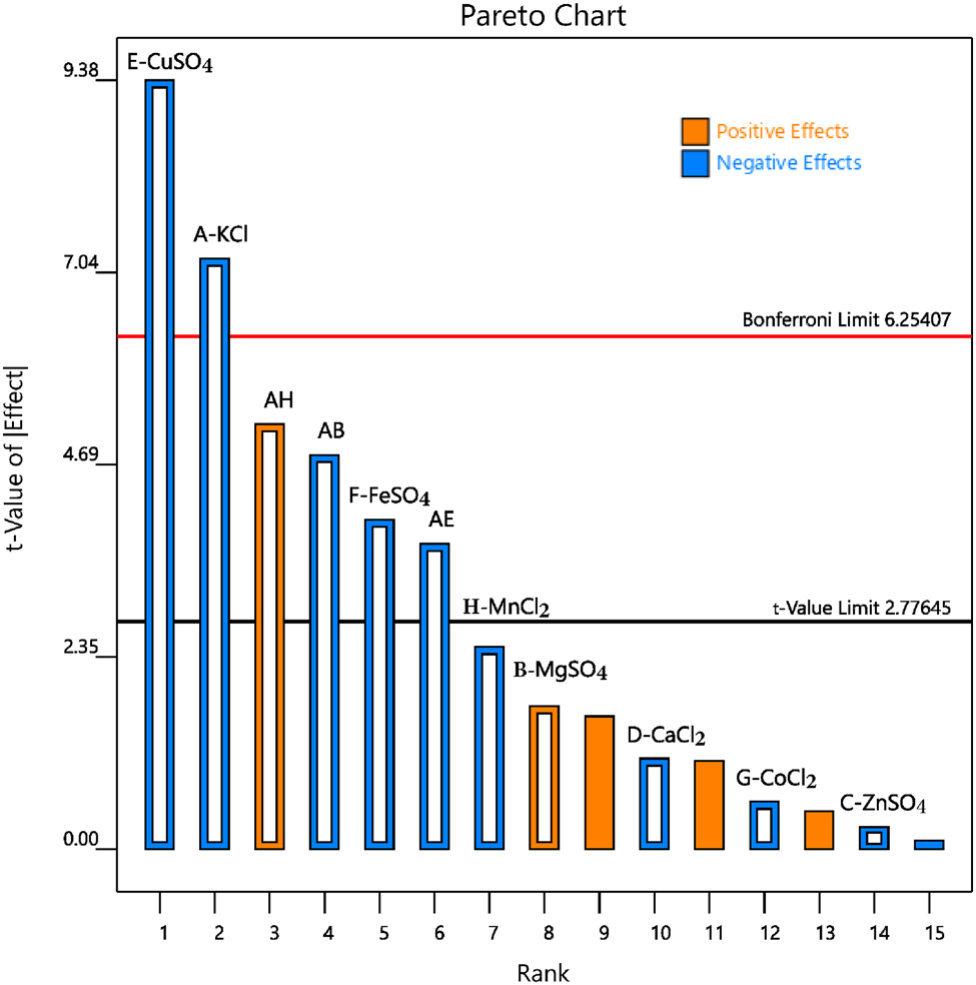
Pareto chart of standardized effects for a two-level fractional factorial design

**Fig. 4:**
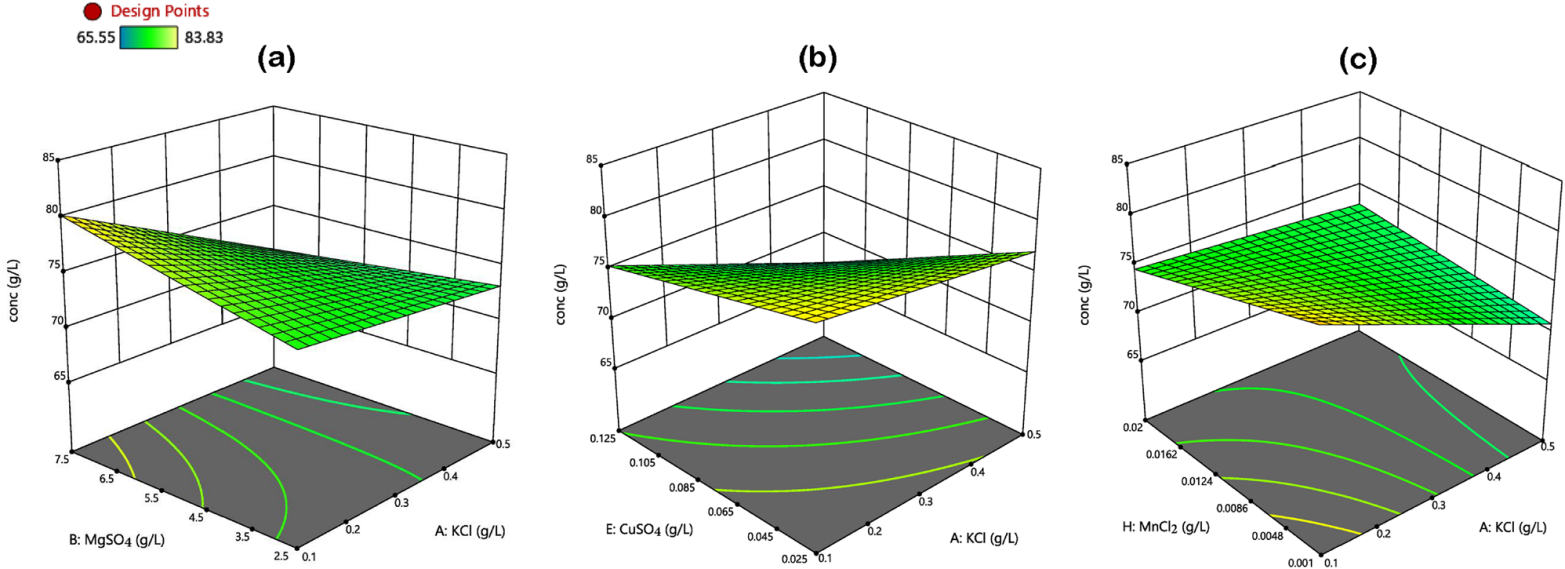
Response surface plots of ethanol concentration as influenced by the interactions of **a** KCl × MgSO_4_, **b** KCl × CuSO_4_, and **c** KCl × MnCl_2_

The Pareto chart for the two-level fractional factorial design shows the standardized main effect of each salt, the 2FI effects, and their order of magnitude (Fig. 3). As indicated in the chart, the main effects of CuSO_4_, KCl, and FeSO_4_ were identified as having significant influences on ethanol concentration, as their t values exceeded the threshold value of 2.78. Whereas, the individual additions of the other salts (MnCl_2_, MgSO_4_, CaCl_2_, CoCl_2_, and ZnSO_4_) made no observable difference on the response variable. Only significant interaction effects were added to the model terms, and they included KCl × MnCl_2_, KCl × MgSO_4_, and KCl × CuSO_4_. According to the employed design, a standardized effect of greater or less than zero depicts a positive or negative effect, respectively. The metal ions screened out for their significant effects (i.e., CuSO_4_, KCl, and FeSO_4_) all had a negative influence on ethanol concentration (Table 7 and Fig. 3). The three-dimensional (3D) surface plots illustrating the response directions of ethanol concentration to the three interaction terms are displayed in Fig. 4. The interaction of MgSO_4_ with KCl had a negative impact on ethanol concentration (Fig. 4a), as maximum response (about 80 g/L) was achievable only at high level of MgSO_4_. Increasing the concentration of KCl progressively caused a significant drop in ethanol titre to the minimum level of about 72 g/L. A similar response trend was also observed in the interaction of CuSO_4_ with KCl (Fig. 4b), whereby the addition of the former initiated a negative response in the dependent variable. In contrast, simultaneously decreasing the input of MnCl_2_ and KCl steadily enhanced ethanol titre to the maximum, which was mutually obtained at the lowest levels of the two salts (Fig. 4c).

Notwithstanding the beneficial effects, a comparative evaluation of the final ethanol titre from the non-supplemented medium (78.04 g/L) and the maximum concentration from nutrient supplemented (83.83 g/L) (as shown in Table 6) revealed no significant difference (*p* < 0.05). The absence of a notable improvement in ethanol concentration and even a significant drop in concentration with the addition of some metallic ions were indications that not only was PMFJ inherently sufficient in yeast-essential minerals (which also agreed with its mineral concentrations as seen in Table 1), but external addition of nutrients might upset the nutrient ion balance in juice, resulting in a reduction of ethanol titre.

Literature reports showed that ethanol production under external supplementation of salt nutrients can vary depending on the employed feedstock. For example, out of diverse metal ions (Fe^2+^, Cu^2+^, Zn^2+^, Mn^2+^, Na^2+^, and Co^2+^) screened for their effect on fermentation of sweet sorghum juice by Cao and Liu (2013), only Mn^2+^ and Co^2+^ were identified as significantly improving final ethanol titre. Pereira et al. (2010) reported that for very high gravity ethanol fermentation using glucose-supplemented corn steep liquor, Mg^2+^ and Cu^2+^ ions were especially critical among other metal ions tested, and thus were isolated for further optimization. On the other hand, Kelbert et al. (2015) observed that the addition of various salts to an already nutrient-sufficient fermentation medium either showed no effect or had a negative effect on ethanol production. The juice of PM exhibited great potentials for utilization in bioethanol production; having met and surpassed some of the conditions for acceptability, including sugar concentration (150–200 g/L), ethanol titre (> 40 g/L), ethanol productivity (> 1 g/L/hr), and fermentation efficiency (> 90%). Added to all these attractive features is the elimination of the need for external nutrient supplementation along with its associated costs, which is an advantage in commercial bioethanol production.

## Conclusions

For the first time, the biotechnological viability and optimal fermentation conditions of the fruit juice of Paper mulberry tree for bioethanol production were successfully evaluated. This sugar and nutrient rich juice offers great promises as a viable feedstock for bioconversion to ethanol, comparing favorably with the juices of typical sugar energy crops. As an important indigenous tree in China, its non-food fruit juice can be usefully exploited in the area of 1G ethanol production, which will add to feedstock diversity, and may thus contribute toward meeting the need for a cleaner, cheaper, and sustainable energy.

### Supplementary Information

**Additional file 1: Table S1.** Residual sugar concentrations (g/L) at various incubation time-points. **Table S2.** Bioethanol production from Paper mulberry fruit juice compared to some notable sugar-based substrates using *S*. *cerevisiae*. **Fig. S1.** Responses of ethanol concentration (top) and productivity (bottom) to the interaction effects of (a) Temperature × Yeast concentration (b) Temperature × pH (c) Yeast concentration × pH. **Fig. S2.** Relationship between residual sugar and ethanol concentration and productivity at 16 hours of fermentation.

## Data Availability

We declare that all data generated or analyzed during this study are included in this published article and its additional file.

## Abbreviations

PM: Paper mulberry
PMFJ: Paper mulberry fruit juice
1G: First generation
DNS: Dinitrosalicylic acid
TRS: Total reducing sugar
HPLC-RI: High-Performance Liquid Chromatography-Refractive Index
RSM: Response surface methodology
