# Supplementary material for: Paper mulberry fruit juice: a novel biomass resource for bioethanol production
Source: Bioresour Bioprocess. 2022 Jan 8;9(1):3. doi: 10.1186/s40643-021-00490-3 (PMC10991237; doi:10.1186/s40643-021-00490-3)
Supplement: Supplementary file 1 — Additional file 1: Table S1. Residual sugar concentrations (g/L) at various incubation time-points. Table S2. Bioethanol production from Paper mulberry fruit juice compared to some notable sugar-based substrates using S. cerevisiae. Fig. S1. Responses of ethanol concentration (top) and productivity (bottom) to the interaction effects of (a) Temperature × Yeast concentration (b) Temperature × pH (c) Yeast concentration × pH. Fig. S2. Relationship between residual sugar and ethanol concentration and productivity at 16 hours of fermentation. [file 40643_2021_490_MOESM1_ESM.docx]

**PAPER MULBERRY FRUIT JUICE: A NOVEL BIOMASS RESOURCE FOR BIOETHANOL PRODUCTION**

Pleasure Chisom Ajayo^a, b^, Mei Huang^a, b^, Li Zhao^a, b^, Dong Tian^a, b^, Qin Jiang^a, b^, Shihuai Deng^a, b^, Yongmei Zeng^a, b^, Fei Shen^a, b,^ ^[[1]](#footnote-1)^*

^a^ Institute of Ecological and Environmental Sciences, Sichuan Agricultural University, Chengdu, Sichuan 611130, P. R. China.

^b^ Rural Environment Protection Engineering & Technology Center of Sichuan Province, Sichuan Agricultural University, Chengdu, Sichuan 611130, P. R. China.

**Table S1 Residual sugar concentrations (g/L) at various incubation time-points**

| Runs | Codes | |  |  | Glucose | Fructose | Total | Glucose | Fructose | Total | Glucose | Fructose | Total | Glucose | Fructose | Total | Glucose | Fructose | Total |
| --- | --- | --- | --- | --- | --- | --- | --- | --- | --- | --- | --- | --- | --- | --- | --- | --- | --- | --- | --- |
|  | XI | X2 | | X3 | **8 h** |  |  | **16 h^*^** |  |  | **24 h** |  |  | **32 h** |  |  | **40 h** |  |  |
| 1 | 30 | 2 | | 4 | 14.72 | 31.82 | 46.54 | 0 | 5.79 | 5.79 | 0 | 5.76 | 5.76 | 0 | 5.8 | 5.8 | 0 | 5.81 | 5.81 |
| 2 | 20 | 0.5 | | 5 | 55.21 | 59.74 | 114.95 | 50.62 | 57.27 | 109.89 | 20.91 | 43.52 | 64.43 | 6.16 | 25.68 | 31.84 | 0 | 7.16 | 7.16 |
| 3 | 30 | 0.5 | | 6 | 34.35 | 49.53 | 83.88 | 0 | 5.96 | 5.96 | 0 | 5.98 | 5.98 | 0 | 5.93 | 5.93 | 0 | 5.9 | 5.9 |
| 4 | 20 | 1.25 | | 6 | 50.49 | 59.27 | 109.76 | 39.45 | 55.40 | 94.85 | 3.76 | 25.03 | 28.79 | 0.36 | 10.98 | 11.34 | 0 | 5.9 | 5.9 |
| 5 | 30 | 1.25 | | 5 | 23.49 | 42.71 | 66.2 | 0 | 6.76 | 6.76 | 0 | 6.73 | 6.73 | 0 | 6.74 | 6.74 | 0 | 6.71 | 6.71 |
| 6 | 30 | 1.25 | | 5 | 24.04 | 44.10 | 68.14 | 0 | 6.79 | 6.79 | 0 | 6.66 | 6.66 | 0 | 6.61 | 6.61 | 0 | 6.62 | 6.62 |
| 7 | 20 | 2 | | 5 | 48.17 | 57.08 | 105.25 | 38.29 | 54.71 | 93.00 | 3.28 | 19.55 | 22.83 | 0 | 7.28 | 7.28 | 0 | 7.06 | 7.06 |
| 8 | 40 | 0.5 | | 5 | 30.27 | 47.61 | 77.88 | 4.98 | 7.83 | 12.81 | 0 | 6.1 | 6.1 | 0 | 6.5 | 6.5 | 0 | 6.2 | 6.2 |
| 9 | 30 | 1.25 | | 5 | 22.08 | 42.37 | 64.45 | 0 | 6.81 | 6.81 | 0 | 6.81 | 6.81 | 0 | 6.84 | 6.84 | 0 | 6.81 | 6.81 |
| 10 | 40 | 1.25 | | 6 | 15.4 | 38.5 | 53.9 | 3.74 | 9.35 | 13.09 | 0 | 6.6 | 6.6 | 0 | 6.57 | 6.57 | 0 | 6.57 | 6.57 |
| 11 | 20 | 1.25 | | 4 | 53.68 | 60.15 | 113.83 | 42.09 | 54.01 | 96.10 | 10.29 | 30.21 | 40.5 | 3.27 | 7.4 | 10.67 | 0 | 7.04 | 7.04 |
| 12 | 30 | 0.5 | | 4 | 39.13 | 52.15 | 91.28 | 2.87 | 3.96 | 6.83 | 0 | 6.81 | 6.81 | 0 | 6.7 | 6.7 | 0 | 6.7 | 6.7 |
| 13 | 40 | 2 | | 5 | 9.98 | 29.98 | 39.96 | 3.19 | 9.58 | 12.77 | 0 | 6.51 | 6.51 | 0 | 6.48 | 6.48 | 0 | 6.48 | 6.48 |
| 14 | 40 | 1.25 | | 4 | 13.95 | 38.16 | 52.11 | 3.41 | 9.31 | 12.72 | 0 | 6.61 | 6.61 | 0 | 6.57 | 6.57 | 0 | 6.59 | 6.59 |
| 15 | 30 | 2 | | 6 | 18.51 | 37.62 | 56.13 | 0 | 5.87 | 5.87 | 0 | 5.87 | 5.87 | 0 | 5.86 | 5.86 | 0 | 5.83 | 5.83 |

*****Evaluations were based on data at this particular time-point

**Table S2 Bioethanol production from Paper mulberry fruit juice compared to some notable sugar-based substrates using *S*. *cerevisiae***

| Feedstocks | Initial total sugar conc. (g/L) | Dominant sugar | Temp. (⁰C) | Yeast conc. (g/L) | pH | Nutrient addition | Time (h) | Ethanol conc. (g/L) | Fermentation efficiency (%) | Ethanol productivity (g/L/hr) | References |
| --- | --- | --- | --- | --- | --- | --- | --- | --- | --- | --- | --- |
| **Paper mulberry fruit juice** | **162** | **Fructose and glucose; 99 %** | **35** | **0.55** | **5** | **Nil** | **16** | **73.7** | **94** | **4.6** | **Current work** |
| Sweet sorghum juice | 95  162 | Sucrose; 45 %  Sucrose; 78 % | 35  37 | 1  12 | 5  4.5 | Nil  Nil | 72  11 | 49.5  72 | 101  87 | 2.4  6.5 | (Luo et al. 2014)  (Barcelos et al. 2016) |
| Sugar cane juice | 230  153–187 | Sucrose  Sucrose; 8–93 % | 30  37 | 20  5 ×10^5^ cells/ml | 5  5 | Nil  Nil | 24  36 | 79.2  9.1 – 10.7 | -  87–90 | 3.3  0.25–0.30 | (Giri et al. 2013)  (Thammasittirong et al. 2017) |
| Sugar beets thin juice concentrate  Sugar beets thick juice  Sugar beets raw juice | 200  210  136 | Sucrose  Sucrose; 99%  Sucrose | 30  30  28 | 1  3  10 | 5  5  5 | Yes  Nil  Nil | 72  46  20 | 91.2  86.3  66.3 | 86  94  94 | 1.3  1.9  4.2 | (Kawa-Rygielska et al. 2013)  (Grahovac et al. 2012)  (Dodić et al. 2012) |
| Banana fruit waste | 485 | - | 35 | 50 | 6 | Yes | 168 | 24.1 | - | - | (Matharasi et al. 2018) |
| Grape fruit waste | - | - | 30 | 10 | 5.6 | Nil | 36 | 58.2 | - | 1.6 | (Dular 2019) |
| Jamaica cherry fruit juice | - | - | 34 | 80 | 6 | Yes | 630 | 74.0 | - | - | (Thangadurai et al. 2014) |


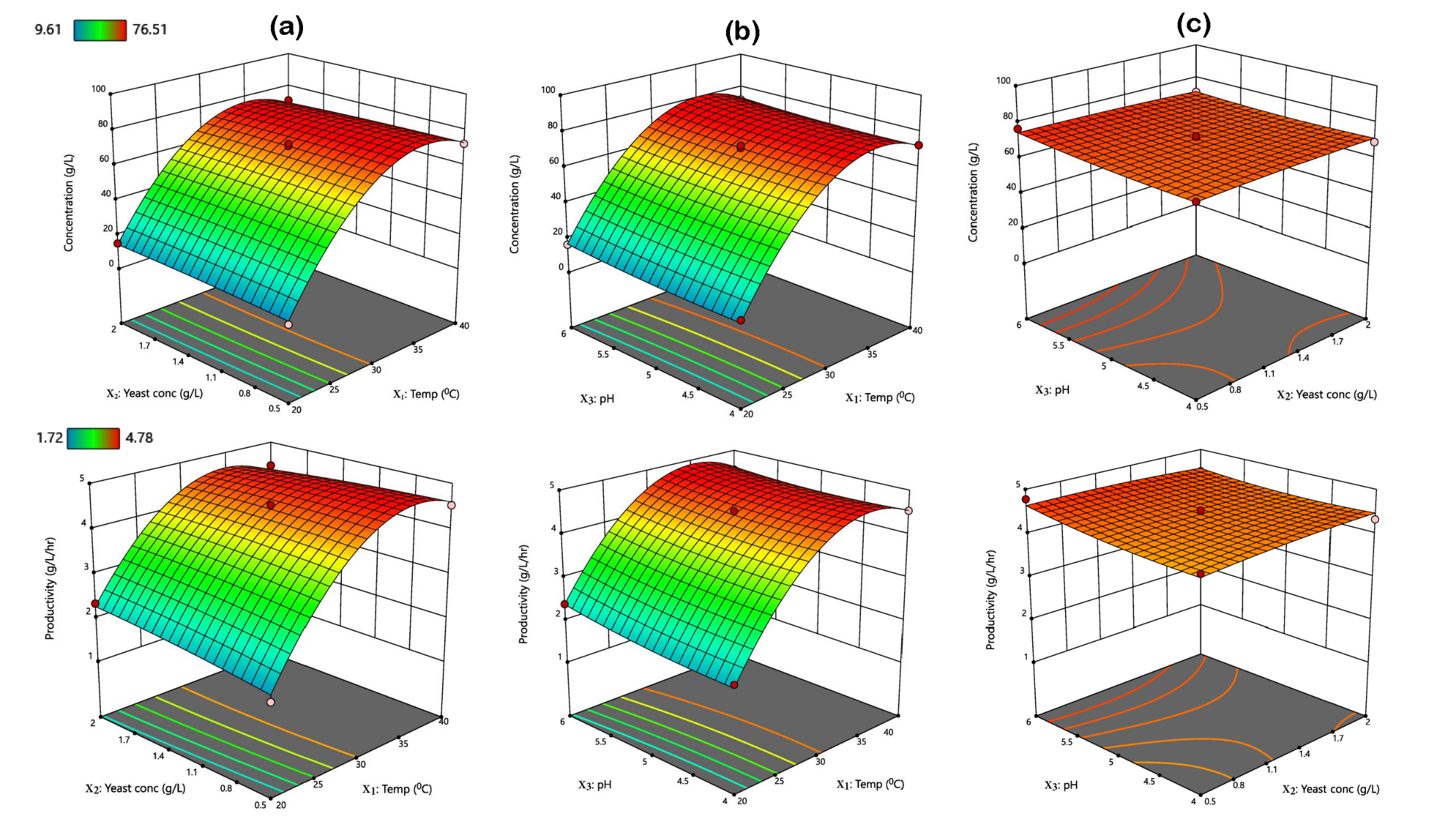


**Fig. S1 Responses of ethanol concentration (top) and productivity (bottom) to the interaction effects of (a) Temperature × Yeast concentration (b) Temperature × pH (c) Yeast concentration × pH**

**Fig. S2 Relationship between residual sugar and ethanol concentration and productivity at 16 hours of fermentation**

**REFERENCES**

Barcelos CA, Maeda RN, Santa Anna LMM, Pereira N (2016) Sweet sorghum as a whole-crop feedstock for ethanol production. Biomass Bioenergy 94:46–56. https://doi.org/10.1016/j.biombioe.2016.08.012

Dodić JM, Vučurović DG, Dodić SN, Grahovac JA, Popov SD, Nedeljković NM (2012) Kinetic modelling of batch ethanol production from sugar beet raw juice. Appl Energy 99:192–197. https://doi.org/10.1016/j.apenergy.2012.05.016

Dular P (2019) Bioethanol Production from Rotten Fruit. Int J Res Appl Sci Eng Technol 7:1555–1560. https://doi.org/10.22214/ijraset.2019.4282

Giri R, Kundu BS, Diwan P, Raj K, Wati L (2013) Ethanol production from direct sugarcane and juice by yeast. Agric Sci Dig - Res J 33:188. https://doi.org/10.5958/j.0976-0547.33.3.005

Grahovac J, Dodić J, Jokić A, Dodić S, Popov S (2012) Optimization of ethanol production from thick juice: A response surface methodology approach. Fuel 93:221–228. https://doi.org/10.1016/j.fuel.2011.10.019

Kawa-Rygielska J, Pietrzak W, Regiec P, Stencel P (2013) Utilization of concentrate after membrane filtration of sugar beet thin juice for ethanol production. Bioresour Technol 133:134–141. https://doi.org/10.1016/j.biortech.2013.01.070

Luo Z, Wang L, Shahbazi A (2014) Optimization of ethanol production from sweet sorghum (Sorghum bicolor) juice using response surface methodology. Biomass Bioenergy 67:53–59. https://doi.org/10.1016/j.biombioe.2014.04.003

Matharasi A, Uma C, Sivagurunathan P, Sampathkumar P (2018) Determination of bioethanol potential from banana waste using indigenous yeast (Saccharomyces cerevisiae KX033583). J Pharmacogn Phytochem 7:2041–2047

Thammasittirong SNR, Chatwachirawong P, Chamduang T, Thammasittirong A (2017) Evaluation of ethanol production from sugar and lignocellulosic part of energy cane. Ind Crops Prod 108:598–603. https://doi.org/10.1016/j.indcrop.2017.07.023

Thangadurai D, Bhat SV., Sangeetha J, Mundaragi AC (2014) Production of Bioethanol from Muntingia calabura Fruits using Saccharomyces cervisiae and Schizosaccharomyces pombe. In: Proceedings of the International Symposium on Agriculture and Environment, University of Rahuna, Sri Lanka, 27 Nov, 2014.

1. * Corresponding author; Address: 211 Huimin Road, Wenjiang District, Chengdu, Sichuan, 611130, P. R. China; Tel. (Fax): +86 28 86293087; E-mail: [fishen@sicau.edu.cn](mailto:fishen@sicau.edu.cn) or [fishensjtu@gmail.com](mailto:fishensjtu@gmail.com) [↑](#footnote-ref-1)
